# Bipolar disorders in DSM-5: strengths, problems and perspectives

**DOI:** 10.1186/2194-7511-1-12

**Published:** 2013-08-23

**Authors:** Jules Angst

**Affiliations:** Research Department, Zurich University Psychiatric Hospital, Lenggstrasse 31, Zurich, 8032 Switzerland

## Abstract

The diagnostic classification of mood disorders by the *Diagnostic and Statistical Manual of Mental Disorders* (DSM-IV-TR) had two major shortcomings: an underdiagnosis of bipolar disorders and a large proportion of treated patients had to be allocated to the vague NOS groups ‘not otherwise specified’. Several new subthreshold groups of depression, bipolar disorders and mixed states are now operationally defined in DSM-5. In addition, hypomanic and manic episodes occurring during antidepressant treatments are, under certain conditions, accepted as criteria for bipolar disorders. The diagnosis of bipolarity now requires, as entry criterion A, not only the presence of elated or irritable mood but also the association of these symptoms with increased energy/activity. This restriction will unfortunately change the diagnoses of some patients from DSM-IV bipolar I and II disorders to subdiagnostic bipolar syndromes. Nonetheless, overall, DSM-5 is a step in the right direction, specifying more subdiagnostic categories with an improved dimensional approach to severity. DSM-5 may also have an impact on patient selection for placebo-controlled drug trials with antidepressants.

## Introduction

The strength of the *Diagnostic and Statistical Manual of Mental Disorders* (DSM-III, DSM-III-R and DSM-IV) was to base psychiatric diagnoses on defined operational criteria, which resulted in high inter-rater reliability. A weakness, shown in relation to DSM-IV, was *that it was only able to formally diagnose under half the patients actually treated* (Angst et al. 2010). This clinically unacceptable situation was derived partly from the lack of operationalized subthreshold diagnoses. Now, in recognition of the fact that for a large group of patients receiving treatment doctors often had no alternative to the residual, catch-all diagnosis not otherwise specified (NOS), DSM-5 includes defined subthreshold syndromes, which will also stimulate research and allow a more dimensional view. For depression, for example, recurrent brief depression and even short-duration depressive episodes (4 to 13 days), as well as 2-week episodes with insufficient symptoms, now have their place.

## Bipolar disorders in DSM-5

The main lines of the DSM-5 definition of major depressive episodes (MDE), basic to the diagnoses of both bipolar I and bipolar II disorders, are similar to those of DSM-IV: presence of five of nine diagnostic symptoms with a minimum duration of 2 weeks and a change from previous functioning. However, it is now possible to specify both depressive disorders and bipolar disorders with mixed features.

The definitions of both manic and hypomanic episodes have been radically revised, which will impact on both bipolar diagnoses. The main changes are three: (1) a problematic change concerning the gate questions (criterion A), (2) a welcome reduction in the number of exclusion criteria and (3) a vigorous effort to operationalize bipolar subthreshold syndromes, hitherto unified under the NOS heading.

### Gate questions for mania and hypomania

Where DSM-IV required, as criterion A, the presence of one of the two mood symptoms (elation/euphoric or irritable mood), in DSM-5, ‘the mood change must be accompanied by persistently increased activity or energy levels’. This new rule is, of course, more restrictive and excludes all individuals who report only one of the three entry symptoms and those with both elated and irritable mood. Thus, for no apparent reason, DSM-5 classifies some patients as having subthreshold bipolar disorders who would formerly have been diagnosed with manic episodes or bipolar I or II disorders. This strict new rule is not based on data, indeed it contradicts available evidence. As the international Bridge Study of 5,635 patients seeking treatment for major depressive episodes demonstrated clearly, any of those three gate questions is valid on its own, according to the criteria established by Robins and Guze (1970) and Angst et al. (2012).

### Exclusion criteria

One important and amply justified change in DSM-5 concerns the diagnosis of bipolar II disorder. In DSM-IV, the change of major depression into *hypomania under antidepressant treatments* (ADs) was in principle an exclusion criterion. In DSM-5, that change - provided it persists at fully syndromal level beyond the physiological effect of the treatment - is explicitly a criterion for bipolar II disorder. DSM-5, like DSM-IV, allows some scope for clinical judgment as to causality. In addition, DSM-5 provides new formal criteria for substance/medication-induced bipolar and related disorder.

On the basis of the Bridge Study data (Angst et al. 2012), we can estimate that DSM-5 bipolar II disorder will be diagnosed about twice as often as heretofore and have a prevalence approaching that of bipolar I.

A more frequent diagnosis of bipolar II disorder is both justified and logical: a milder condition (in this case hypomania) is usually more prevalent than a severe one (mania). Over the long-term course of their illness, bipolar patients spend much more time in milder conditions, mainly minor depression, than in major syndromes (Phillips and Kupfer 2013).

Two exclusion criteria survive in DSM-5, namely ‘substance/medication-induced bipolar and related disorder’ and ‘bipolar and related disorder due to another medical condition’. Both clearly rely on questionable causal attributions based on partial co-occurrence with substance or medication use or full co-occurrence with another medical condition.

### Other specified bipolar and related disorder (DSM-5)

DSM-5 has fortunately replaced DSM-IV's vague group NOS by defining MDE with several subthreshold conditions of bipolarity, for instance, allowing a duration of 2 to 3 days for hypomanic episodes, as suggested by child psychiatrists, or fewer than four symptoms of hypomania during 4 days, or, for cyclothymia, specifying shorter manifestations (<24 months). A further important step is the recognition that dysthymia can co-occur with hypomania which is considered as a co-morbid condition, but why - one might ask - is it not allocated to cyclothymic disorder?

## Underdiagnosis of bipolar disorders, hypomania and mania

The underrecognition of bipolar disorder is sadly set to continue despite the advances of DSM-5 described above. The re-analyses of large epidemiological studies demonstrated that DSM major depressive disorder (MDD) is clearly heterogenous and includes about 40% of hidden bipolars. Without systematic screening for hypomania in patients' previous history, DSM-5 will have little appreciable impact on the detection of this hidden bipolarity. The vast majority of patients with MDE will continue to be diagnosed as having MDD.

In this context, DSM-5's (and ICD's) non-recognition of pure mania and hypomania as diagnostic entities remains problematic in view of the accumulating evidence. Both conditions are fairly common in adolescence (Päären et al. 2013). Moreover, the large, representative, epidemiological NCS-A study (*N* = 10,123 adolescents aged 13 to 18 years) has demonstrated the frequent independence of mania and hypomania from depression (Merikangas et al. 2012). Most recently, the NIMH family study of patients with mood disorders has shown that mania is even genetically independent (Merikangas et al., in press). Adolescents, unlike adults, more often meet the DSM-IV criteria for mania and hypomania without MDD than for bipolar disorders, but they are often unaware of their mood changes, whereas adults' retrospective assessments are rich in false negatives, as Moffitt et al. (2010) have recently demonstrated in relation to major depressive episodes.

With the predicted continuing underdiagnosis of bipolar disorder, the underprescription of lithium, its best established prophylactic treatment, is also likely to persist. Lithium reduces suicides, improves the course of the illness and may even lower the risk of dementia in these patients (Angst et al. 2007; Nunes et al. 2007; Kessing et al. 2008), whose risk of dementia is elevated (da Silva et al. 2013).

## Recommendations for trials with antidepressants

Non-response to ADs in MDD is correlated with hidden bipolarity (Hantouche et al. 2009; Rybakowski et al. 2010; Correa et al. 2012). Systematic screening for hypomanic symptoms during the selection of patients for controlled antidepressant trials would have several benefits. It would identify bipolarity in patients with major depressive episodes and increase the homogeneity of the samples, increase the responder rates and the power of placebo-controlled trials, and finally reduce the sample sizes required. Systematic measures of hypomanic symptoms by rating scales during the trials would help to identify the development of mixed states and switches into hypomania.

## Future directions in research on the bipolar spectrum

As I see it, future research should focus on the independence of mania and hypomania from bipolar disorder, and the unsolved issue in adolescent psychiatry of whether hyperthymic behaviour in some adolescents remains within the normal range of variation of emotional development or emotional dysregulation (Päären et al. 2013). This developmental phase is strongly associated with the start of substance misuse (tobacco, alcohol and drugs), which may be secondary to normal adolescent ‘highs’ or to early hypomanic episodes, as suggested by the results of the NCS-A study (see also review of Post and Kalivas 2013). Adolescents therefore pose a special difficulty - that of distinguishing between developmental trait/temperament (hyperthymia) and states (hypomanic or mixed episodes). The traditional criteria for caseness, such as distress or impairment, are not applicable to typical syndromes of hypomania and mania since the subjects do not feel in any way ill or impaired. In most cases, the only conclusive basis for diagnosing undesired social consequences may be the information provided by parents, friends, teachers or employers.

Another topic requiring further research is the duration criteria for MDE (2 weeks) and for hypomania (4 days), the validity if which has been questioned by recent data from the Bridge and the Zurich studies (Angst et al. 2012). In principle, all continuous variables, such as distress/suffering, impairment, episode duration and time spent in illness over 1 year (2 years for a chronic syndrome), should be measured systematically in clinical assessments and not just dichotomized for diagnostic definitions.

Structured diagnostic interviews for clinical and epidemiological purposes should include all subthreshold categories (‘other specified diagnostic categories 311’ (F32.8) and ‘other specified bipolar and related disorder’ 296.89 (F31.89). This can provide the necessary data for future revisions.

Urgently needed, but underfunded, are methodologically sound prospective studies of patient and community samples, taking both somatic and psychiatric aspects of health and illness equally into account. Here, the new DSM-5 will certainly help, but it would be short-sighted to restrict data collection to current diagnostic concepts, which will have a short half-life of 10 years or less. Other perspectives for future biological research on the spectrum of unipolar depression and bipolar disorder have been outlined by Phillips and Kupfer (2013).
